# Empyema thoracis presented as giant back abscess

**DOI:** 10.1016/j.radcr.2021.02.030

**Published:** 2021-02-24

**Authors:** Siti Hafzan Abd Karim, Wan Zainira Wan Zain, Mohd Nizam Mohd Hashim, Andee Dzulkarnaen Zakaria, Firdaus Hayati, Chiak Yot Ng

**Affiliations:** a Department of General Surgery, Hospital Universiti Sains Malaysia, School of Medical Sciences, Universiti Sains Malaysia, Kota Bharu, Kelantan, Malaysia; b Department of Surgery, Faculty of Medicine and Health Sciences, Universiti Malaysia Sabah, Kota Kinabalu, Sabah, Malaysia; c Department of Medicine, Faculty of Medicine and Health Sciences, Universiti Malaysia Sabah, Kota Kinabalu, Sabah, Malaysia

**Keywords:** Abscess, Bacterial infection, Pleural empyema, Thoracic empyema

## Abstract

Empyema thoracis (ET) is defined as pus in the pleural space, either localized or involving the entire pleural cavity, due to diverse etiologies. In severe form, it may infiltrate the extrapulmonary region. Clinical guideline describes 3 stages of parapneumonic effusion before developing into an ET, namely the exudative stage, the fibrinopurulent stage, and the organizing/late stage. We highlight a 59-year-old gentleman who presented a back swelling masquerading as a deep-seated abscess, in which the diagnosis of ET had not been established early. The principles of treatment are treating the underlying condition such as pneumonia, pleural drainage and debridement, full re-expansion of the collapsed lung by performing chest physiotherapy, and improving nutrition. ET is a condition with a dynamic process due to diverse etiologies, either localized or involving the entire pleural cavity. The gold standard in diagnosing ET is the pleural aspiration of fluid from the pleural space, whereas the management of ET may include non-surgical and/or surgical treatments based on the basic principles of ET treatment.

## Introduction

Pleural effusion is among the complications of pneumonia. While antibiotics lead to disease resolution in most patients, some patients develop a more fibrinous reaction leading to frank pus in the most severe cases. The latter is referred to as an empyema thoracis (ET). The incidence has been gradually increasing, despite advancements in the era of antibiotics treatment. Mortality and morbidity vary between 3% and 33% [1]. Therefore, by avoiding parapneumonic effusion from becoming empyema through prompt evaluation and aggressive intervention, reduction in morbidity, mortality, and the overall healthcare cost can be achieved [2].

ET is a condition with a dynamic process due to diverse etiologies, either localized or involving the entire pleural cavity [2]. Diseases in the pleura or the lung can lead to the development of primary empyema meanwhile the secondary thoracic empyema results from a previous intervention to the chest [3]. Among these, the most common forms of ET are post parapneumonic effusions and thoracic surgical procedures [2]. ET is mostly suspected upon detection of pyrexia of unknown origin in individuals with respiratory symptoms, and rarely detected from extrapulmonary manifestations. We highlight a 59-year-old gentleman who presented a back swelling masquerading as a subcutaneous abscess, in which the diagnosis of ET had not been established early.

## Case description

**Fig. 1:**
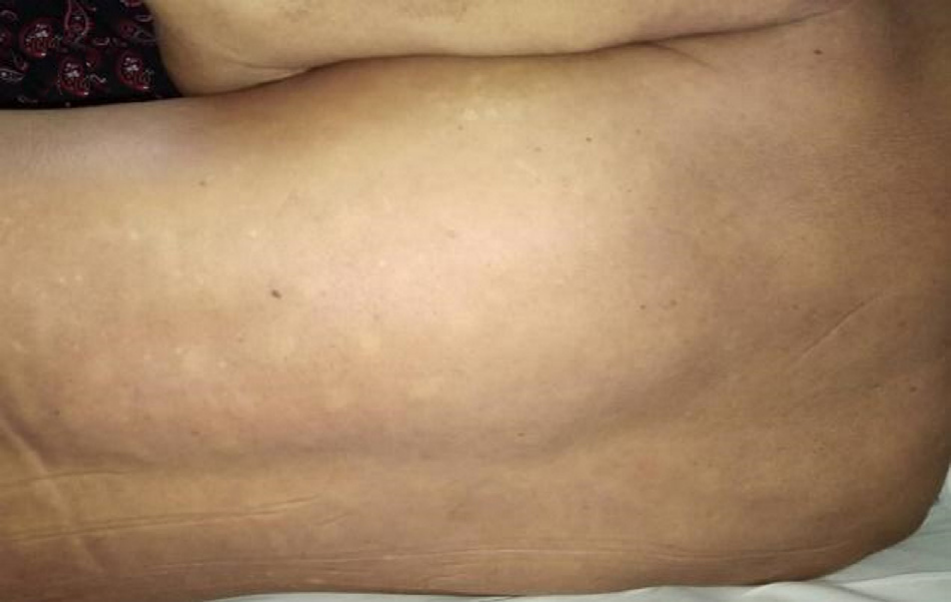
The posterior view of the bulging area (*) over the left side of the thoracic region.

A 59-year-old male presented pain and swelling over the left posterior back, associated with chesty cough, lethargy, and poor oral intake for 1 week. He denied any trauma or insect bite to the back. He was recently diagnosed with Type II Diabetes and was started on oral anti-hyperglycemic agent. On presentation, though he was in sepsis, with tachycardia (heart rate of 110 bpm) and febrility (temperature of 39.2°C), there was no hypotension. There was decreased air entry over the left lung from the base up to the midzone. Abdominal examination was normal. Instead, there was a huge swelling (Fig. 1) occupying almost half of his left posterior torso, with overlying erythematous skin and subcutaneous crepitus. It was tender and fluctuated upon palpation.

**Fig. 2:**
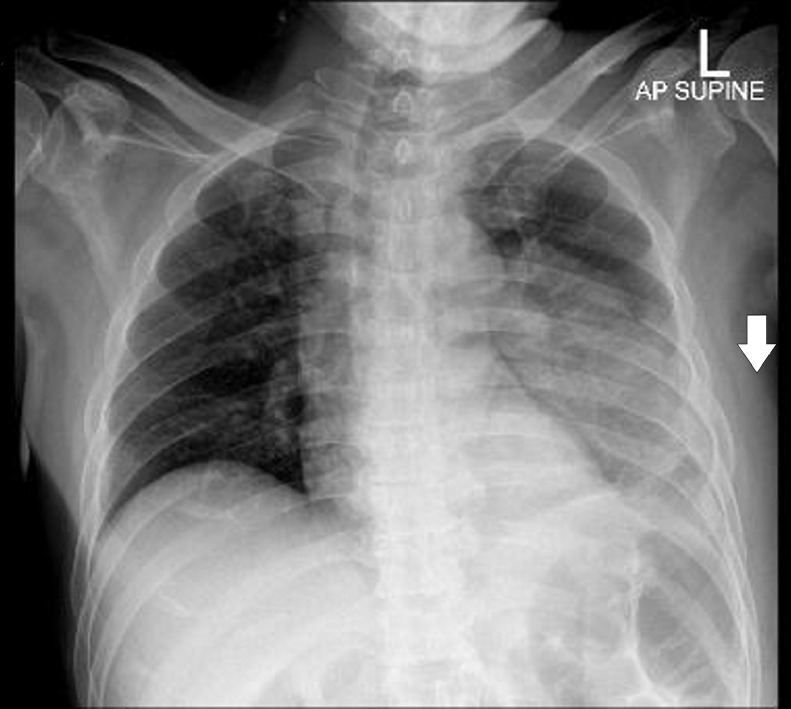
Chest radiograph of the initial presentation, showing consolidation occupying the middle and the lower zone of the left lung with left pleural effusion; thickening of the left thoracic wall's soft tissue (white arrow).

Blood investigation showed markedly-raised white blood cells of 25 (normal value: 7-11 × 10^9^), hyponatremia of 121 (normal value: 135-400 mmol/L), and hyperkalemia of 5.2 (normal value: 3.5-4.0 mmol/L). However, there was no acidosis. Initial assessment led towards a subcutaneous abscess. Chest radiography (Fig. 2) showed consolidation at the middle and the lower zones of the left lung, with pleural effusion and the presence of soft tissue opacity which had caused thickening of the left thoracic chest wall. A thoracic ultrasound was performed with suspicion of a probable subcutaneous abscess. The thoracic ultrasound revealed an intramuscular collection at the posterior part of the left thorax, extending into the subcutaneous tissue.

**Fig. 3:**
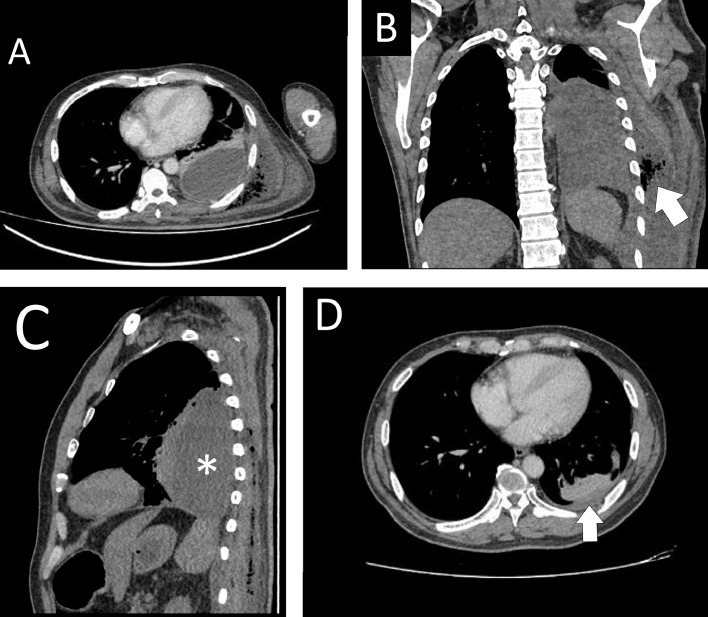
(A) Enhanced-contrast CT of the thorax at the axial view, showing a hypodensity in the left pleural cavity (*) with an extensive subcutaneous, extrathoracic, gas-forming extension (white arrow) of the collection to the left posterior chest wall, suggestive of left ET. (B) The collection with gas-forming extension (white arrow) at the coronal view. (C) The collection (*) at sagittal view. (D) A repeat CT of the thorax, revealing a reduction of the left pleural effusion (white arrow) and left posterior chest wall collection after completing 2 weeks of antibiotics.

Considering that the patient has a left-sided pleural effusion and an intramuscular collection on the left thoracic wall, there is a possibility of empyema thoracis where these 2 collections communicated. Hence, a computed tomography (CT) scan of the thorax (Fig. 3A) was arranged, which finally confirmed a left-sided ET with extensive left posterior back subcutaneous collection starting from level T1 to level L4 of the vertebrae. Two size-12 Fr pigtail drainage catheters (Dawson-Mueller Drainage Catheters, Cook Medical Interventional Radiology, UK) were inserted separately: one to drain the left lung empyema and the other to drain the left posterior back collection. Pus culture grew *Streptococcus dysgalactiae*; biochemistry of pus reported no protein detection, and a complete biochemical test was unable to proceed due to very thick fluid. The cytology result showed an exudative fluid with prominent degenerated cells, necrotic debris, and bacterial colonies. Tuberculosis (TB) work-up was negative, C-reactive protein was 200 (normal value: <3 mg/L) on presentation. Throughout admission, the patient recovered well in the ward, with a repeated ultrasound showing a size reduction of the collection. The pleural drain was removed and the patient was discharged with a left subcutaneous pigtail catheter. Upon follow up, he showed recovery, with resolution of the ET as evidenced by a repeat CT of the thorax (Fig. 3B).

## Discussion

The development of parapneumonic effusions and empyema occurs into 3 stages over a period of 3 to 6 weeks [3]. The 3 stages are described as 1: the exudative stage; 2: the fibrinopurulent stage; and 3: the organizing/late stage. In the description of the last and worst stage, pus becomes extremely thick and progressive granulation occurs. In the event that empyema is not well treated, pus may spontaneously seep out through the surrounding structures including the chest wall, tracheobronchial tree, or mediastinal structures [4]. The patient in this case report showed a stage-3 ET, observed from the empyema already extending into the subcutaneous tissue, despite being localized in the pleural cavity which might have started with an effusion but was complicated later due to late intervention.

The most common clinical presentations are unilateral sharp chest pain on inspiration, cough, dyspnea, and high-grade pyrexia. Usually there is a preceding history of an insult such as lower respiratory tract infection (40%), recent thoracic instrumentation (40%) with incomplete recovery, or underlying trauma (10%) [4]. During the initial attack, patients often will be pyrexic, tachycardic, tachypnic, and have decreased air entry on auscultation. There will be a basal dullness on percussion with overlying tenderness [4]. The later stages will feature a significant loss of weight, poor nutrition, reduction in chest excursion, and pronounced dyspnea. Fibrothorax eventually occurs with complete pulmonary encasement and severe thoracic cavity constriction [4]. These may correlate with our patient's similar presentation. However, instead of solely pulmonary symptoms, he presented extrapulmonary manifestation, which perplexed us and prompted us to intervene.

A chest radiography may reveal pleural thickening, effusion, or air-fluid levels [2,3]. Basal opacity or meniscus on the plain chest radiography can be visualized in the early stages of empyema. Ultrasound is the most sensitive modality to stage the empyema, evaluate the fibrin content of a pleural collection, and demonstrate septations [4]. In addition, physicians commonly use ultrasound to mark the site of drainage or to enable the guided drainage insertion [3]. CT is used to evaluate the location and size of pleural collections [4]. It is also useful to rule out malignancy, differentiate pleural empyema from parenchymal lung abscess, and guide intervention and operation planning [3].

Comprehensive evaluation, rapid diagnosis and holistic treatment of ET are crucial toward achieving satisfactory outcomes and minimizing morbidity [4]. The principles of treatment are treating the underlying condition such as pneumonia, pleural drainage and debridement, full re-expansion of the collapsed lung by performing chest physiotherapy, and improving nutrition [2,3]. Apart from the general supportive measures, appropriate antibiotics are delivered based on the results from gram stain culture and sensitivity [2]. Drainage via the insertion of a thoracostomy tube is necessary when the pleural fluid is purulent, turbid, or cloudy in appearance. It is more effective in exudative effusions and in the fibrinopurulent stage. In our patient, pigtail drainage catheters were inserted and resulted in the drainage of pus and cloudy fluid. Fibrinolytic therapy is a non-surgical option in the management of ET or exudative pleural effusion [5]. By choosing this option, it allows an intrapleural instillation of fibrinolytic agents that can breakdown the septa and increase the efficacy of chest tube drainage [6].

Surgical management is warranted when antibiotic therapy and chest drainage have failed to achieve resolution. A bronchoscopy is mandatory prior to surgery to rule out endobronchial obstruction from the inspissated sputum, inhaled foreign material, or tumor [3]. Video-assisted thoracic surgery is a minimally invasive procedure providing a better visualization for a more effective debridement [6]. However, the conversion to an open thoracotomy is inevitable in the events of incomplete drainage or failed expansion of the lung [6]. Open surgery allows the best access and tactile assessment of the chest cavity, but is associated with significantly more postoperative pain and respiratory morbidity. When patients present with an advanced empyema on a background of prohibitive comorbidity and malnourishment, a rib resection and long-term open drainage are advisable to achieve the promising outcomes.

In conclusion, ET is a condition with a dynamic process due to diverse etiologies, either localized or involving the entire pleural cavity. The gold standard in diagnosing ET is the pleural aspiration of fluid from the pleural space, whereas the management of ET may include non-surgical and/or surgical treatments based on the basic principles of ET treatment.

## Informed consent

Written informed consent was obtained from the patient for the publication of this case report.

## Author contributions

SHAK prepared and wrote this article. WZWZ and MNMH involved in managing the patient besides preparing the figures. ADZ contributed in the editing the final manuscript. FH wrote and revised the manuscript as well as acted as the corresponding author. CYN involved in providing the radiological descriptions. All authors have access to the manuscript and had agreed to publish as in the current form.
